# Cortical and Subcortical Signatures of Incentive Salience Attribution in Tobacco Use Disorder

**DOI:** 10.1111/adb.70151

**Published:** 2026-05-17

**Authors:** Nicola Sambuco, Francesco Versace, Brian A. Taylor

**Affiliations:** ^1^ Department of Translational Biomedicine and Neuroscience University of Bari Aldo Moro Bari Italy; ^2^ Department of Behavioral Science The University of Texas MD Anderson Cancer Center Houston Texas USA; ^3^ Department of Imaging Physics The University of Texas MD Anderson Cancer Center Houston Texas USA

**Keywords:** cue reactivity, emotional pictures, fMRI, incentive salience, individual differences, sign‐tracking, tobacco use disorder

## Abstract

Relapse is a defining feature of substance use disorders, yet relapse risk varies widely across individuals. This variability has been linked to individual differences in incentive salience attribution to drug‐related cues. We used fMRI to identify cortical and subcortical circuits underlying individual differences in incentive salience attribution among people with tobacco use disorder. Fifty daily cigarette users viewed pleasant, unpleasant, cigarette‐related and neutral images during task‐based fMRI and completed a resting‐state scan. We extracted per‐condition BOLD activity from a functionally defined extended visual region (Emotional > Neutral) and applied *k*‐means clustering (*k* = 2) to derive neuroaffective profiles. Whole‐brain voxelwise tests assessed Group × Content (cigarette vs. pleasant) effects. We compared groups' resting‐state connectivity at the network level and for subcortical–cortical pairs. Individuals were clustered based on neuroaffective reactivity to drug‐related and pleasant stimuli: The C > P profile showed larger BOLD responses to cigarette cues than to pleasant stimuli, whereas the P > C profile showed the opposite pattern. Whole‐brain analyses showed significant Group × Content interactions that mirrored these profiles across left dorsolateral and medial prefrontal cortices and amygdala–hippocampal regions. At rest, individuals in the C > P profile showed weaker within‐network connectivity of the frontoparietal control network (FPCN) and, in exploratory analyses, reduced FPCN–nucleus accumbens coupling. By replicating and extending prior psychophysiological work, this study identifies neuroaffective profiles that reflect the variation in incentive salience attribution central to neurobehavioral models of relapse. These findings provide convergent neurobiological markers that characterize distinct motivational pathways in substance use disorders.

## Graphical abstract

Using fMRI, we identified two neuroaffective profiles in daily cigarette smokers based on BOLD reactivity to cigarette‐related versus pleasant cues in the extended visual system. The sign‐tracker‐like profile (C > P) showed enhanced responses to cigarette cues in prefrontal and amygdala–hippocampal regions, along with weaker resting‐state frontoparietal control network connectivity. These convergent task‐based and resting‐state markers provide neurobiological evidence for individual differences in incentive salience attribution relevant to relapse vulnerability.

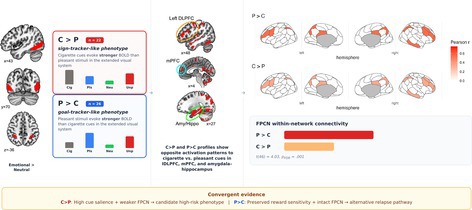

## 1 Introduction

Relapse is a defining feature of substance use disorders (SUDs) and can occur even after prolonged periods of abstinence [1]. Neurobehavioral models of addiction posit that relapse vulnerability stems from dysregulation in the neural mechanisms that control incentive salience attribution [2, 3]. Incentive salience refers to the motivational properties of rewards and the cues associated with them [4]. The ability to flexibly attribute incentive salience to reward‐related cues is adaptive because it allows individuals to efficiently detect, prioritize and pursue rewards. However, when drug‐related cues acquire disproportionately high incentive salience, they can control behaviour, acting as powerful triggers for compulsive drug seeking and relapse [5]. Preclinical findings show that some animals, called sign‐trackers, are particularly susceptible to compulsive cue‐induced behaviours (including cue‐induced drug self‐administration) because they have the tendency to assign high incentive salience to reward‐related cues. In contrast, goal‐trackers assign lower incentive salience to cues and are more resistant to cue‐induced behaviours [6, 7]. These findings have clear translational implications: If similar psychophysiological differences exist in patients with SUDs, they may become a therapeutic target to reduce relapse vulnerability. Hence, identifying a biomarker of the tendency to attribute high incentive salience to drug‐related cues is a critical next step to enable risk stratification and guide the development of personalized interventions to reduce relapse.

To replicate sign‐tracking in humans, many researchers have measured attentional bias to reward‐related cues using behavioural measures such as reaction times or eye movements [8]. Consistent with animal models, results showed that some individuals continue to show attentional bias toward reward‐related cues even when the cues become task‐irrelevant [9, 10]. However, this approach neither linked individual differences in sign‐tracking to clinical outcomes in SUDs nor clarified the neurophysiological mechanisms underlying these differences. To address these gaps, we adopted a neurophysiological approach to directly measure the incentive salience individuals attribute to both drug‐related and non‐drug‐related motivationally relevant cues. In this context, measuring brain responses to non‐drug‐related cues is critical, as these stimuli provide a benchmark for determining the motivational relevance of drug‐related cues [11]. As a measure of motivational relevance, we used the amplitude of the late positive potential (LPP), a well‐validated component of the event‐related potentials (ERPs) [12, 13]. Using a data‐driven clustering approach, we identified two neuroaffective profiles: one characterized by stronger LPP responses to drug‐related than to pleasant cues (C > P), the other showing the opposite pattern (P > C) [14]. We interpreted these profiles as reflecting individual differences in the incentive salience attributed to drug‐related cues: Individuals with the C > P profile attribute high incentive salience to drug‐related cues, whereas individuals with the P > C profile assign relatively low incentive salience to the same cues. Importantly, we showed that these profiles are clinically significant: Individuals with the C > P profile show stronger attentional bias toward drug‐related cues [15], are more prone to cue‐induced nicotine self‐administration [16] and are more likely to relapse when attempting to quit smoking [17] than those with the P > C profile.

While ERPs measure brain activity with high temporal resolution, they cannot reliably disentangle cortical and subcortical contributions to neuroaffective responses [12, 18, 19]. This limitation is critical because the circuits primarily involved with reward responses, incentive salience attribution and addictive behaviours include subcortical regions, such as the ventral striatum and other limbic structures [20, 21]. Therefore, in the current study, we used functional magnetic resonance imaging (fMRI) to investigate how cortical and subcortical systems contribute to individual differences in neuroaffective reactivity to drug‐related and non‐drug‐related cues in individuals with tobacco use disorder. First, we determined the reproducibility of the C > P and P > C neuroaffective profiles using fMRI (Blood‐Oxygen‐Level‐Dependent) activity from the extended visual system, the neural generators of the LPP [22]. Next, we tested whether individuals assigned to the two profiles also show differential activation in subcortical and prefrontal regions implicated in incentive salience attribution and whether they show distinct patterns of intrinsic functional connectivity [23]. Linking cue‐reactivity profiles to large‐scale network properties [24] will refine our understanding of the psychophysiological mechanisms underlying relapse vulnerability in people with SUDs and will inform the development of personalized interventions aimed at reducing cue‐induced relapse.

## 2 Methods

### 2.1 Participants

All procedures were approved by the Institutional Review Board of The University of Texas MD Anderson Cancer Center. Data were collected from January 2022 to July 2024. Adults were eligible if they were 21–65 years old, reported smoking ≥ 5 cigarettes/day and provided biochemical verification of smoking via a positive cotinine test on the day of the session. Exclusion criteria included a self‐reported history of major psychiatric disorders (e.g., depression, bipolar disorder, schizophrenia and PTSD), high alcohol use (> 14 drinks/week), current treatment for substance use, significant sensory impairments, a history of seizures or epilepsy, concussion with loss of consciousness within the past 6 months, standard MRI contraindications (e.g., MR unsafe implants, claustrophobia) and, if female, pregnancy (confirmed via urine test on the day of the visit). All participants provided written informed consent prior to study procedures.

We recruited participants by advertising the study on social media, distributing flyers and through word of mouth. Fifty‐one participants completed the study. Of these, two had only partial data acquisition due to equipment failure, and one had more than 40% of TRs censored due to excessive motion (the other participants had a maximum of 7% of censored TRs, with an average across the entire sample of 0.5%). Data from these participants were excluded, resulting in a final sample of 48 participants.

### 2.2 Data Acquisition

Participants were scanned on a Siemens 3T Prisma System (Malvern, PA). Structural MRI was acquired with a 3D T1‐weighted magnetization prepared rapid gradient echo (MPRAGE) sequence (TE/TR/TI = 2.98/2300/900 ms, 25.6 × 25.6 cm^2^ FOV with a 256 × 256 matric, 9° flip angle and 1.2‐mm slice thickness). Task‐based fMRI was acquired with a gradient echo‐echo planar imaging (GRE‐EPI) sequence with two sequential acquisitions of 340 volumes each (TE/TR = 30/2000 ms, 19.2 × 19.2 cm^2^ FOV with a 64 × 64 matrix and 3‐mm slice thickness). Resting‐state data were acquired with a GRE‐EPI sequence of 180 volumes (TE/TR = 25/2000 ms; 24.0 × 24.0 cm^2^ FOV with a 64 × 64 matrix and 4‐mm slice thickness).

### 2.3 Picture Viewing Task and Resting‐State MRI

Participants completed a picture‐viewing task comprising 72 images drawn from the International Affective Picture System (IAPS) [25] and from laboratory smoking‐related picture sets [26]. Images were organized into six semantic categories (12 images per category): pleasant–erotica, pleasant–romantic, unpleasant–mutilation, unpleasant–violence, neutral (individuals engaged in everyday activities) and cigarette‐related (people smoking). (Stimulus identifiers: Cigarette [CIG; none of the images were selected from the IAPS]. Neutral [Neu]: 2102, 2107, 2191, 2305, 2377, 2393, 2396, 2397, 2411, 2500, 2575, 9070. Pleasant—high arousal [Erotica; ‘PH’]: 4604, 4611, 4658, 4659, 4668, 4677, 4687, 4691, 4800 [plus three images not selected from the IAPS]. Pleasant—low arousal [Romantic; ‘PL’]: 4597, 4612, 4624, 4625, 4640, 4641, 4643 [plus five images not selected from the IAPS]. Unpleasant—high arousal [‘UH’]: 3030, 3051, 3053, 3060, 3069, 3100, 3120, 3170, 3225, 3261, 3400, 9253. Unpleasant—low arousal [‘UL’]: 3500, 3530, 6231, 6242, 6313, 6315, 6540, 6550, 6571, 9429‐2, 9520 [plus one image not selected from the IAPS].) Images were presented in a pseudorandom order with the constraint that no more than two images from the same category appeared consecutively. Each image was displayed for 4 s, followed by a jittered intertrial interval of 16, 18 or 20 s with a central fixation cross. Stimuli were shown on an fMRI‐compatible monitor at the end of the scanner bore and viewed via a head‐coil–mounted mirror. Participants were instructed to view each image for its entire presentation, remain still and minimize head and body movement.

The resting‐state scan was conducted approximately 15 min after the task‐based fMRI. Participants were instructed to remain awake, keep their eyes open and focus on a fixation cross presented on the screen, relax, minimize movement and avoid structured thoughts.

### 2.4 Functional MRI Data Processing

#### 2.4.1 Picture Viewing

Preprocessing was conducted in AFNI [27] and included slice‐timing correction, head‐motion correction (realignment to a reference volume), coregistration to each participant's anatomical scan, brain masking, spatial smoothing, intensity scaling and quality control. A binary brain mask derived from the anatomical image was resampled to functional space. Functional volumes were spatially smoothed with a 6‐mm FWHM Gaussian kernel and scaled to percent‐signal change relative to each voxel's mean. Quality control comprised visual inspection and automated outlier detection. To mitigate motion artefacts, time points where the fraction of outlier voxels exceeded 0.05 (5%) were censored from subsequent analyses. Outlier voxels were identified via AFNI's 3dToutcount, which flags voxels whose signal at a given time point deviates substantially from the local temporal trend based on median absolute deviation [27, 28]. This threshold resulted in minimal data loss (mean across participants: 0.5% of TRs; maximum: 7%).

Hemodynamic responses were estimated with a voxelwise general linear model (GLM) using AFNI's deconvolution framework. Task regressors (one per condition) were modelled with a canonical gamma function using event onsets from condition‐specific timing files. Six motion parameters (roll, pitch, yaw; and translations along the superior–inferior, left–right and anterior–posterior axes) were included as nuisance regressors. Volumes flagged for excessive motion/outliers were censored. Models were estimated via ordinary least squares, and resulting contrast/statistical maps were normalized to MNI152 space and resampled to 3‐mm isotropic resolution for group analyses.

#### 2.4.2 Resting State

Preprocessing removed head motion (six parameters: x, y, z, roll, pitch, yaw) and baseline/drift effects (second‐order polynomial). Volumes with excessive artefacts were identified with 3dToutcount and censored (overall removal < 2% across participants). Time series were normalized to MNI152 space and resampled to 3‐mm isotropic resolution. For each participant, we extracted BOLD time series from 100 cortical ROIs defined by the Schaefer–Yeo atlas [29] and 16 subcortical ROIs derived from the Tian atlas [30]. As for the task‐based analyses, all resting‐state analyses have been conducted in AFNI.

### 2.5 Data Analysis

#### 2.5.1 Voxel‐Wise ANOVA

We conducted a voxelwise mixed‐effects ANOVA in AFNI to identify regions sensitive to emotional content. A two‐factor model included Condition (six categories) and Subject (random effect). A planned contrast tested Emotional (cigarette, pleasant, unpleasant) > Neutral. Familywise error was controlled via 3dClustSim using AFNI's mixed ACF model; with a voxelwise threshold of *p* < 0.001 (uncorrected), simulations indicated a minimum cluster size of 50 voxels for α = 0.05 (cluster‐level). Notably, the Emotional > Neutral effect during picture viewing shows good–excellent replicability in lateral occipito‐temporal cortex (and amygdala), supporting this contrast as the basis for downstream ROI definition [31].

#### 2.5.2 Classification of Participants via Clustering

To classify participants, we extracted per‐condition beta estimates from the extended visual system ROI defined by the Emotional > Neutral map above (an orthogonal definition with respect to cigarette vs. pleasant differences). Betas were *z*‐transformed within subject to emphasize profile shape over overall amplitude and then submitted to *k*‐means clustering (*k* = 2). Based on our prior work [14], we expected the profiles to reflect individual differences in the incentive salience of the drug‐related cues: One group (C > P) would show larger BOLD responses to drug‐related than to pleasant cues and the other (P > C) showing the opposite reactivity profile.

#### 2.5.3 Whole‐Brain Group Differences Outside the Visual ROI

To localize differences beyond the ROI used for clustering, we computed a Cigarette − Pleasant difference image for each participant and ran a voxelwise independent‐samples *t*‐test comparing C > P versus P > C groups excluding the clustering ROI from the search space. Multiple comparisons were controlled using 3dClustSim (ACF model; voxelwise *p* < 0.001, cluster‐level α = 0.05).

#### 2.5.4 Resting‐State Connectivity Analyses

For each participant, ROI time series were pairwise Pearson‐correlated and Fisher *r*‐to‐*z* transformed. Within‐network connectivity for the seven Schaefer–Yeo canonical networks (visual, somatomotor, control, dorsal attention, ventral attention/salience, limbic and default mode) was computed as the mean of all Fisher‐*z* edges among parcels within each network. Group differences (C > P vs. P > C) were tested at the network level; *p* values were controlled across the seven tests using FDR (*q* = 0.05). For subcortical–cortical connectivity, we first averaged left/right time series to create bilateral signals for eight subcortical ROIs [30]. For each subcortical ROI, we then computed its mean Fisher‐*z* correlation with all parcels in each cortical network, yielding an 8 × 7 (subcortex × network) summary matrix per participant. This bilateral averaging (which exploits the high interhemispheric correspondence of subcortical nuclei) and network‐level averaging reduce the number of statistical tests and stabilize estimates by shrinking edge‐level noise. Group differences on the 8 × 7 summaries were assessed with independent‐samples *t*‐tests and FDR‐controlled across all comparisons (*q* = 0.05).

## 3 Results

### 3.1 Participants

Table 1 shows the characteristics of the sample: an ethnically diverse group in their mid‐to‐late 40s, roughly evenly split by sex. Participants reported moderate nicotine dependence as indicated by a mean FTND score of 4.08 (SD = 2.27) [39] and moderate craving levels (QSU total *M* = 43.64, SD = 24.69). Self‐reported depression and anxiety symptoms were low and below clinical cutoffs. Trait impulsivity, assessed via the BIS‐11, was within normal ranges across all three subscales (attentional, motor and nonplanning) [40]. The two subgroups did not differ significantly on any demographic, smoking‐related or mood‐related characteristic (all *p*s > 0.23).

**TABLE 1:** Demographic, smoking‐related and mood‐related characteristics of participants in Cluster 1 (P > C; *N* = 26) and Cluster 2 (C > P; *N* = 22), as well as the full sample (*N* = 48).

	Cluster 1 (P > C) *N* = 26	Cluster 2 (C > P) *N* = 22	All participants *N* = 48	Group comparison P > C vs. C > P
Demographics, *M* (SD)	*M* (SD)	*M* (SD)	*M* (SD)	DF = 46
Age (years)	47.7 (11.3)	46.3 (13.6)	47.0 (12.3)	*t* = −0.37, *p* = 0.708
Body mass index (BM)	30.3 (8.3)	28.9 (6.5)	29.6 (7.5)	*t* = 0.92, *p* = 0.363
Females (*N*)	53%	41%	48	*χ* ^2^ = 1.40, *p* = 0.237
Race (%)				
Black or African American	54	36	46	
Native Hawaiian/Pacific Islander	4	0	2	
White	31	55	42	
More than one race	7	9	8	
Other/I prefer not to say	4	0	2	
Ethnicity (%)				
Non‐Hispanic/Latino	81	77	79	
Hispanic/Latino	19	18	19	
Other/I prefer not to say	0	5	2	
Smoking‐related self‐reports, *M* (SD)				
FTND	4.19 (2.25)	3.95 (2.34)	4.08 (2.27)	*t* = −0.16, *p* = 0.876
QSU	46.20 (24.01)	40.62 (25.69)	43.64 (24.69)	*t* = −0.916, *p* = 0.365
Mood‐related self‐reports, *M* (SD)				
GAD7	2.50 (2.40)	2.41 (3.40)	2.46 (2.87)	*t* = −0.04, *p* = 0.964
PHQ9	3.23 (2.79)	3.32 (3.51)	3.27 (3.11)	*t* = −0.11, *p* = 0.914
Panas NA	15.96 (7.28)	15.59 (6.56)	15.79 (6.89)	*t* = −0.21, *p* = 0.834
Panas PA	34.19 (7.32)	36.45 (6.92)	35.23 (7.15)	*t* = −0.18, *p* = 0.855
SHAPS	5.27 (5.10)	5.50 (4.93)	5.38 (4.97)	*t* = 0.15, *p* = 0.875
BIS11 attentional score	13.00 (3.84)	12.95 (2.95)	12.98 (3.42)	*t* = −0.13, *p* = 0.896
BIS11 motor score	21.54 (5.47)	21.36 (3.29)	21.46 (4.56)	*t* = −0.40, *p* = 0.691
BIS11 nonplanning score	22.65 (5.75)	22.05 (4.57)	22.38 (5.20)	*t* = 1.09, *p* = 0.280

*Note:* Values are presented as means (SD) or percentages. Group comparisons between clusters were conducted using independent‐sample *t*‐tests for continuous variables and *χ* ^2^ tests for categorical variables. Measures include the Fagerström Test for Cigarette Dependence (FTCD) [32], the Questionnaire of Smoking Urges (QSU) [33], the Generalized Anxiety Disorder 7‐item scale (GAD‐7) [34], the Patient Health Questionnaire (PHQ‐9) [35], the Positive and Negative Affect Schedule (PANAS) [36], the Snaith–Hamilton Pleasure Scale (SHAPS) [37] and the Barratt Impulsiveness Scale (BIS‐11) [38].

### 3.2 Functional Regions Activated During Emotional Picture Viewing

**FIGURE 1:**
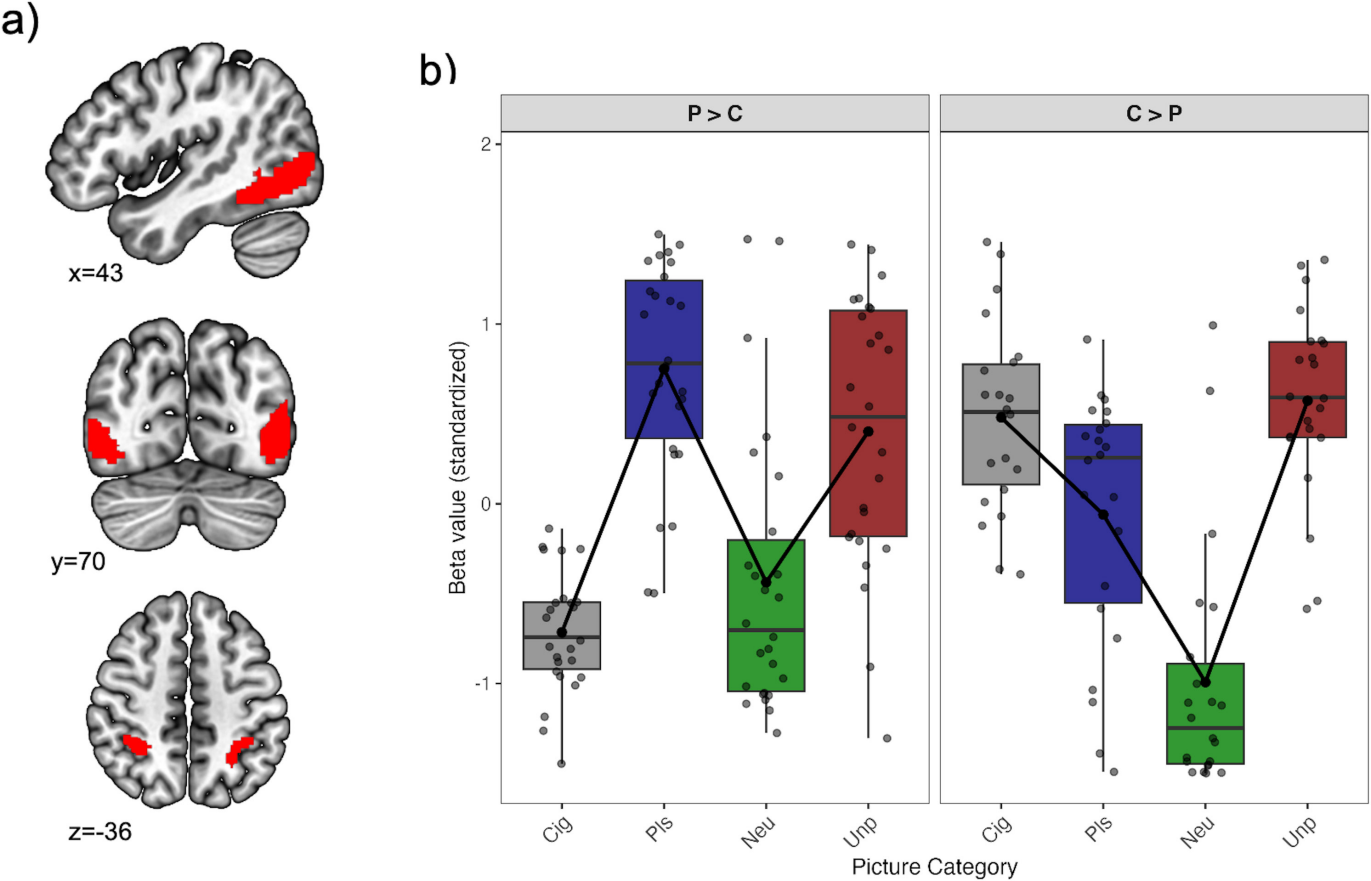
Emotional scenes robustly engage lateral occipito–temporal/parietal cortex; activity within this extended visual ROI segregates smokers into two neuroaffective profiles that differ in relative reactivity to cigarette versus non‐drug emotional images. (a) Functional regions for Emotional > Neutral (voxelwise *p* < 0.001; pFDR < 0.033), encompassing lateral occipito–temporal and inferior/superior parietal cortex. This extended visual/parietal ROI was used for profile derivation. (b) Beta estimates (conditions: Cig = cigarette‐related; Pls = pleasant; Neu = neutral; Unp = unpleasant) within the two k‐means clusters from (a). P > C profile: Pls/Unp > Cig/Neu (both *p* < 0.001). C > P profile: Cig ≈ Pls/Unp > Neu (all *p* < 0.001 vs. Neu).

As illustrated in Figure 1a (see also Table 2), the contrast between emotional and neutral conditions identified four significant functional regions (*p* < 0.001 uncorrected, pFDR < 0.033). The first functional region (11 063 voxels) was primarily located in the left middle temporal gyrus (33.9% overlap), with additional involvement of the left middle occipital gyrus (28.1%), the left inferior occipital gyrus (17.7%) and the left inferior temporal gyrus (11.0%). The second functional region (8849 voxels) was primarily located in the right inferior temporal gyrus (35.3% overlap), with additional contributions from the right middle temporal gyrus (28.0%), the right inferior occipital gyrus (17.5%) and the right middle occipital gyrus (11.7%). The third functional region (2610 voxels) was localized to the left inferior parietal lobule (64.1%) and the left superior parietal lobule (35.5%). The fourth functional region (1928 voxels) was identified in the right inferior parietal lobule (56.4%) and the right superior parietal lobule (16.0%). For subsequent analyses, BOLD signal and beta estimates were averaged across all functional regions.

**TABLE 2:** In line with the ‘highlighting’ approach to results reporting [41], this table summarizes the spatial extent of significant clusters by listing their overlap with multiple anatomical regions, rather than reducing each cluster to a single peak coordinate.

Functional region	Size	Overlap	ROI location
Contrast: Emotional versus neutral (*p* < 0.001, pFDR < 0.033)			
1	11 063	33.9%	Left_Middle_Temporal_Gyrus
		28.1%	Left_Middle_Occipital_Gyrus Left
		17.7%	Inferior_Occipital_Gyrus
		11.0%	Left_Inferior_Temporal_Gyrus
2	8849	35.3%	Right_Inferior_Temporal_Gyrus
		28.0%	Right_Middle_Temporal_Gyrus
		17.5%	Right_Inferior_Occipital_Gyrus
		11.7%	Right_Middle_Occipital_Gyrus
3	2610	64.1%	Left_Inferior_Parietal_Lobule
		35.5%	Left_Superior_Parietal_Lobule
4	1928	56.4%	Right_Inferior_Parietal_Lobule
		16.0%	Right_Superior_Parietal_Lobule
Contrast: Group (Group 1, Group 2) × picture category (pleasant, cigarette‐related) (*p* < 0.001, pFDR < 0.038)			
1	2405	9.60%	Right_Hippocampus
2	822	46.6%	Right_Middle_Occipital_Gyrus
		32.6%	Right_Superior_Occipital_Gyrus
3	822	53.2%	Left_Middle_Occipital_Gyrus
		17.9%	Left_Superior_Occipital_Gyrus
		14.7%	Left_Superior_Parietal_Lobule
4	818	37.7%	Left_Fusiform_Gyrus
		23.4%	Left_Lingual_Gyrus
		16.9%	Left_Inferior_Temporal_Gyrus
5	729	27.2%	Right_Cerebellum_(VIII)
		18.2%	Left_Cerebellum_(IX)
		15.9%	Right_Cerebellum_(IX)
		11.4%	Left_Cerebellum_(VIII)
6	511	51.3%	Right_Lingual_Gyrus
		47.7%	Right_Fusiform_Gyrus
7	347	86.2%	Right_Middle_Temporal_Gyrus
		10.2%	Right_Superior_Temporal_Gyrus
8	206	45.6%	Right_Superior_Temporal_Gyrus
		38.5%	Right_Rolandic_Operculum
		11.6%	Right_SupraMarginal_Gyrus
9	204	60.9%	Left_Postcentral_Gyrus
		20.4%	Left_Inferior_Parietal_Lobule
		16.2%	Left_Superior_Parietal_Lobule
10	193	56.0%	Right_Superior_Parietal_Lobule
		44.0%	Right_Precuneus
11	187	69.9%	Right_Superior_Temporal_Gyrus
		29.6%	Right_Middle_Temporal_Gyrus
12	162	66.5%	Right_Inferior_Temporal_Gyrus
		27.5%	Right_Middle_Temporal_Gyrus
13	131	31.8%	Left_Cerebellum_(Crus_2)
		16.6%	Left_Cerebellum_(VIII)
14	129	69.1%	Left_Middle_Temporal_Gyrus
		23.7%	Left_Inferior_Temporal_Gyrus
15	125	59.3%	Left_Precentral_Gyrus
		40.7%	Left_Middle_Frontal_Gyrus
16	117	72.60%	Right_Inferior_Temporal_Gyrus
17	95	64.2%	Left_Inferior_Occipital_Gyrus
		25.4%	Left_Middle_Occipital_Gyrus
18	93	53.1%	Right_Calcarine_Gyrus
		46.9%	Right_Precuneus
19	93	40.5%	Right_Middle_Cingulate_Cortex
		28.5%	Right_Paracentral_Lobule
		21.1%	Right_Precuneus
20	80	62.6%	Left_Middle_Temporal_Gyrus
		37.2%	Left_Superior_Temporal_Gyrus
21	76	97.90%	Left_Inferior_Frontal_Gyrus_(p.*Triangularis)
22	733	36.0%	Left_Cerebellum*(VIII)
		19.5%	Left_Cerebellum_(VII)
23	58	90.30%	Right_Postcentral_Gyrus
24	57	44.9%	Right_Cerebellum_(IV–V)
		26.4%	Right_Cerebellum_(III)
		17.0%	Cerebellar_Vermis_(3)
25	52	53.40%	Right_Insula_Lobe

*Note:* For each cluster, the table details its size in voxels size (number of voxels) and the percentage of its volume that overlaps with specific Regions of Interest (ROIs) derived from a standard anatomical atlas provided in AFNI (MNI_caez_ml_18). This method provides a more complete and stable representation of cluster locations, showing instances where activation spans several distinct anatomical areas. All reported clusters passed a statistical threshold of *p* < 0.001.

### 3.3 Neuroaffective Classification Based on Brain Responses to Cigarette‐Related and Emotional Pictures

*Z*‐transformed beta estimates from the extended visual/parietal ROI (Figure 1a) were entered into *k*‐means (*k* = 2) cluster analysis to derive neuroaffective profiles (Figure 1b). Replicating our ERP‐based results [42, 43], both profiles showed larger BOLD responses to emotional than neutral cues (all *p*s < 0.005) but differed in reactivity to the cigarette‐related cues: One profile (P > C; *N* = 26) was characterized by greater responses to pleasant images than cigarette cues (*p* < 0.001) while the other (C > P; *N* = 22) showed stronger responses to cigarette cues than pleasant images (*p* < 0.02) (Figure 1b). These results indicate that both groups processed intrinsically pleasant and unpleasant stimuli as motivationally relevant, but only individuals in the C > P group attributed relatively high salience to cigarette‐related cues.

### 3.4 Whole‐Brain Group Differences for Cigarette Versus Pleasant Contents

**FIGURE 2:**
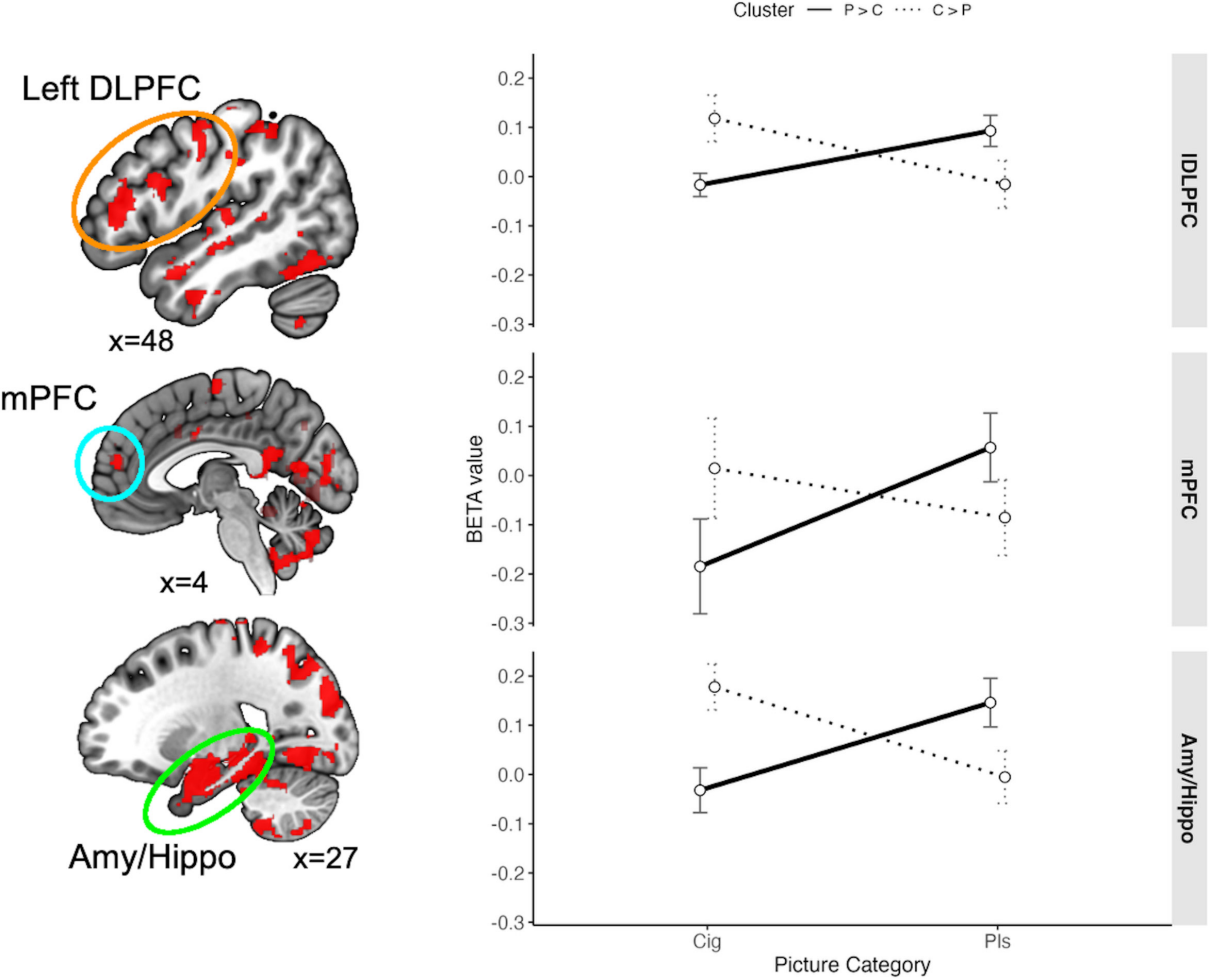
Regions showing a significant group (Group 1, Group 2) × picture category (pleasant, cigarette‐related) interaction in a voxelwise generalized linear model conducted across all voxels that were not used to divide participants into groups. The right panel illustrates the interaction pattern in three functional regions identified in the interaction, the left dorsolateral prefrontal cortex (lDLPFC), the medial prefrontal cortex (mPFC) and the amygdala‐hippocampal regions (Amy/Hippo). Bars indicate the 95% CI.

Whole‐brain analyses showed a widespread Group (P > C, C > P) × Content (pleasant, cigarette) interaction (voxelwise *p* < 0.001, cluster‐corrected α = 0.05; Figure 2; Table 2). The pattern was consistent across regions including medial prefrontal cortex (mPFC), left dorsolateral prefrontal cortex (lDLPFC) and amygdala–hippocampal areas: mPFC *F*(1, 46) = 8.72, *p* = 0.005, *ηp* ^2^ = 0.159; lDLPFC *F*(1, 46) = 21.77, *p* < 0.001, *ηp* ^2^ = 0.321; amygdala–hippocampal *F*(1, 46) = 36.66, *p* < 0.001, *ηp* ^2^ = 0.259. Paired sample *t*‐tests showed P > C exhibited pleasant > cigarette responses in lDLPFC (*t* = 3.378, *p* = 0.002), mPFC (*t* = 2.884, *p* = 0.008) and amygdala–hippocampal regions (*t* = 4.171, *p* < 0.001). Conversely, C > P showed cigarette > pleasant in lDLPFC (*t* = 3.340, *p* = 0.003) and amygdala–hippocampal regions (*t* = 4.306, *p* < 0.001), with no differences detected in mPFC (*t* = 1.557, *p* = 0.134). Full regional details are provided in Table 2.

Exploratory cross‐sectional correlations between ROI reactivity and clinical measures revealed several nominally significant associations (supplementary results and Figure S1), including relationships between amygdala–hippocampal reactivity and anhedonia in the P > C profile. However, these did not survive correction for multiple comparisons and should be interpreted cautiously given the modest sample size.

### 3.5 Resting‐State

#### 3.5.1 Functional Connectivity in Canonical Cortical Networks

**FIGURE 3:**
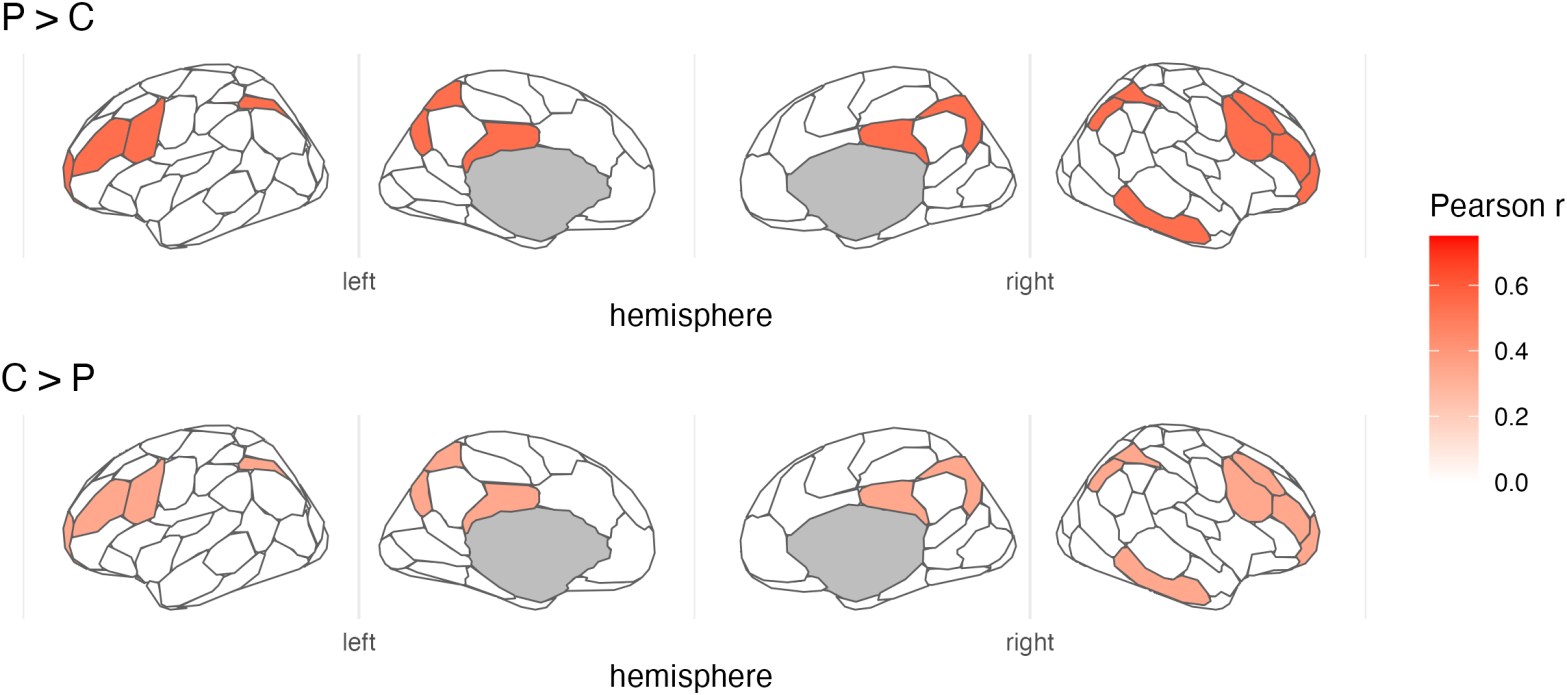
Individuals with high drug cue reactivity (C > P) show reduced intrinsic connectivity within the Fronto‐Parietal Control Network. Glass brains show correlation values for two distinct clusters, reporting these values only in the regions of the control network in which the group effect survived false discovery rate (FDR) correction. The top panel displays the results for P > C, and the bottom panel shows the results for C > P. For all other networks not meeting this significance threshold, the correlation values have been artificially set to zero. The colour scale indicates the strength of the correlation (Pearson *r*), with warmer colours representing higher values.

Our primary resting‐state connectivity analysis examined whether network‐level functional connectivity within canonical large‐scale cortical networks differed between the two neuroaffective clusters. As shown in Figure 3 (see also Table 3), connectivity patterns differed significantly between the two clusters for the fronto‐parietal control network, *t*(46) = 4.033, *p* < 0.001, pFDR = 0.001, with individuals in the P > C cluster showing stronger connectivity than those assigned to the C > P. No significant differences were found for the dorsal attention, default mode, limbic, somatomotor, salience and ventral attention or visual networks after FDR correction (all pFDR > 0.70).

**TABLE 3:** Differences in functional connectivity between clusters across large‐scale brain networks.

Network	*t*(46)	*p* (unc)	pFDR
Dorsal attention	−0.046	0.96	0.99
Default mode	0.0685	0.95	0.99
**Control**	**4.033**	**0.0002**	**0.0014**
Limbic	0.011	0.99	0.99
Somatomotor	−1.302	0.20	0.70
Salience and ventral attention	−0.035	0.97	0.99
Visual	−0.111	0.91	0.99

*Note:* Independent samples *t*‐tests were conducted to compare connectivity strength between the two clusters across seven intrinsic connectivity networks: dorsal attention, default mode, control, limbic, somatomotor, salience and ventral attention and visual networks. The only effect that remains significant after false discovery rate (FDR) correction is presented in bold.

#### 3.5.2 Functional Connectivity Between Subcortical Regions and Cortical Networks

**FIGURE 4:**
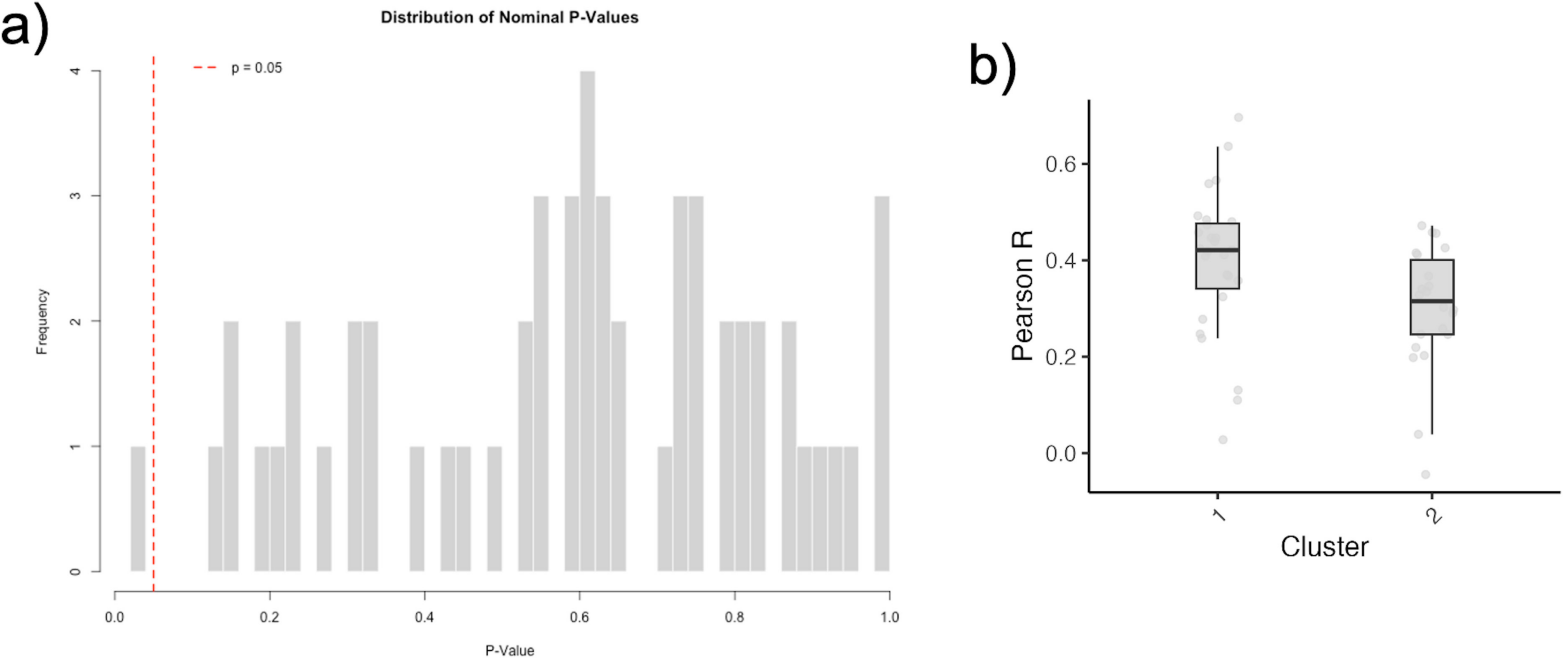
Exploratory analysis showed reduced connectivity between the nucleus accumbens and the control network in the high cue‐reactive group (C > P). Results of the subcortical–cortical connectivity analysis of resting‐state fMRI. (a) Distribution of nominal *p*‐values from 56 independent *t*‐tests. The red dashed line indicates the significance threshold (*p* = 0.05). (b) Boxplot showing the difference in connectivity (Pearson *r*) between the nucleus accumbens and the control network that reached nominal significance but did not survive FDR correction. Note: Cluster 1: P > C; Cluster 2: C > P.

To investigate whether individuals assigned to the two neuroaffective clusters based on the picture‐viewing task differed in subcortical–cortical functional connectivity during resting‐state fMRI, we conducted exploratory analyses on the connectivity between eight subcortical regions and seven cortical networks, yielding a correlation matrix of 56 values. Among those connections (Figure 4a), only one was below the nominal threshold of *p* < 0.05, but none of them survived FDR correction. Specifically, as illustrated in Figure 4b, the connectivity between the nucleus accumbens and the control network was lower in individuals in the C > P cluster compared to those in the P > C cluster, *t*(46) = 2.320, *p* = 0.025. Because this effect did not survive FDR correction, we consider it as an exploratory, hypothesis‐generating observation rather than a primary result.

## 4 Discussion

This study integrated task‐based and resting‐state fMRI to identify neurobiological profiles associated with individual differences in the attribution of incentive salience to drug‐related cues among people with tobacco use disorder. We found that individual differences in neuroaffective responses to drug‐related versus non‐drug‐related motivational stimuli cluster according to two profiles. Using ERPs, we have previously demonstrated that these profiles are associated with differential vulnerability to cue‐induced drug self‐administration and relapse risk [16, 43]. Notably, convergence between our prior ERP findings and the current fMRI results was evident not only in the patterns of neuroaffective reactivity but also in the absence of group differences in demographic and self‐reported characteristics. This cross‐modality convergence supports our interpretation that these neuroaffective profiles reflect a stable phenotype rather than a modality‐specific artefact. The two neurobehavioral profiles conceptually align with those observed in preclinical models of sign‐ and goal‐tracking behaviour, where individual differences in the incentive salience that animals assign to reward‐predictive cues underlie susceptibility to cue‐induced compulsive reward seeking, including drug seeking [44]. Hence, our approach provides a translational framework for better understanding the neurobiological underpinnings of maladaptive, cue‐induced behaviours in humans and for informing the development of personalized treatments for substance use disorders.

### 4.1 Task‐Based fMRI Uncovers Profiles of Incentive Salience Attribution

To benchmark the motivational relevance of cigarette cues, we used BOLD responses from the visual system during the presentation of a wide range of non‐drug‐related emotional stimuli. The emotional > neutral BOLD response is a reliable neuromarker of motivational relevance, with high cross‐sample replicability [31]. Unlike conventional cue‐reactivity paradigms that include only drug‐related and neutral images [45], our paradigm directly contrasts cigarette cues with potent non‐drug‐related affective stimuli. This methodological shift is critical for accurately assessing the salience of drug‐related cues while simultaneously evaluating the integrity of both the appetitive and defensive motivational systems [12] and it is in line with recent work showing that emotional experiences are represented in distributed networks that include visual cortex as well as supramodal prefrontal regions [46, 47]. In fact, our results indicate that while the two profiles showed marked differences in neuroaffective reactivity to drug‐related cues, both showed intact reactivity to emotional stimuli, supporting the internal validity of our results. By grounding our classification in this fundamental neuroaffective response, our paradigm directly captures individual differences in the attribution of incentive salience, rather than infer them from behavioural tasks aimed at reproducing sign‐tracking behaviours in humans [7, 8]. Using fMRI also allowed us to better characterize the cortical and subcortical circuits associated with these profiles, setting the stage for developing personalized mechanism‐informed relapse‐prevention interventions.

### 4.2 Neurobiological Phenotypes Shows Distinct Pathways in Addiction

#### 4.2.1 The C > P Phenotype: High Cue Salience Meets Weaker Cognitive Control

Task‐based and resting‐state findings converge on a candidate high‐risk phenotype. Individuals who attribute high incentive salience to cigarette cues (C > P) also show reduced within‐network connectivity of the frontoparietal control network (FPCN) at rest and, in exploratory analyses, a nominally weaker FPCN–nucleus accumbens coupling, although this effect did not survive FDR correction. The FPCN has been implicated in goal‐directed control and regulation across multiple tasks, and the nucleus accumbens in reward processing; thus, one hypothesis is that this pattern may index a vulnerability to cue‐driven behaviour. However, because the present study did not include task‐based measures of cognitive control or emotion regulation, the functional significance of these resting‐state differences remains inferential and should be regarded as hypothesis‐generating. Although FPCN hypoconnectivity has been observed broadly in substance use [48, 49] and reduced executive control is central to the Impaired Response Inhibition and Salience Attribution (iRISA) model [5], our results indicate that these network alterations are not uniformly present across individuals with SUDs. Instead, they are more prominent in the C > P subgroup, consistent with preclinical evidence indicating that sign‐trackers are characterized by both stimulus‐driven processing and weaker cortical control [50]. Notably, this neurally defined profile did not emerge from self‐reports of trait impulsivity, suggesting that the vulnerability captured by the C > P profile may be context‐specific, emerging in the presence of drug‐related cues rather than reflecting a generalized lack of control. If replicated, this dissociation would highlight the limitations of self‐reports and underscore the value of neuroimaging for identifying biologically grounded subtypes that can guide circuit‐targeted interventions.

#### 4.2.2 The P > C Phenotype: A Different Path to Addiction

The P > C group shows a more adaptive neuroaffective profile: stronger responses to natural rewards than to cigarette cues, along with relatively preserved FPCN integrity and FPCN–accumbens coupling. Why, then, do these individuals relapse after a quit attempt? For them, internal states such as nicotine withdrawal, stress reactivity, or negative affect, rather than the hyper‐salience of external cues, may be the primary drivers of smoking [51, 52]. Importantly, the P > C profile is not characterized by blunted reward responsivity. On the contrary, its preserved sensitivity to non‐drug rewards and intact executive control circuitry stands in contrast to reports of generalized reward blunting in some SUD samples [20, 53]. These findings support a resilience perspective: lower drug‐cue salience and preserved valuation of alternative rewards. Identifying biomarkers of these alternate pathways to relapse is a priority for developing truly personalized interventions.

#### 4.2.3 Cerebellar Involvement in Neuroaffective Profiles

Beyond prefrontal and limbic structures, the Group × Content interaction also involved cerebellar regions VIII and IX. The cerebellum has traditionally been viewed as primarily involved in motor coordination, but accumulating evidence implicates posterior cerebellar lobules in cognitive and affective processing relevant to addiction [54, 55, 56]. Specifically, lobules VIII and IX—part of the ‘cognitive cerebellum’—show robust functional connectivity with prefrontal and limbic networks and have been associated with executive control, error monitoring, and habit formation [57, 58]. In the context of substance use disorders, cerebellar involvement has been linked to cue reactivity, craving, and the transition from goal‐directed to habitual drug seeking [55]. The differential engagement of these regions in our neuroaffective profiles may reflect distinct patterns of cognitive‐motor circuit recruitment during the processing of motivationally salient stimuli. However, because our study was not designed to specifically interrogate cerebellar function, we consider this finding hypothesis‐generating and encourage future work to examine the cerebellar contribution to individual differences in cue reactivity and addiction vulnerability.

### 4.3 Implications for Mechanism‐Informed, Personalized Interventions

While the cross‐sectional design of this study limits conclusions about individual‐level stratification and treatment efficacy, our findings nonetheless suggest testable hypotheses for mechanism‐informed interventions. For individuals in the C > P profile, who show heightened cue reactivity and lower frontoparietal control network connectivity, future work could test whether treatments that reduce cue salience and/or strengthen executive control confer particular benefit. Noninvasive neuromodulation offers complementary levers: stimulation of the ventromedial prefrontal cortex could reduce cue salience and craving [59, 60], and stimulation of the dorsolateral prefrontal cortex could enhance FPCN function and top‐down regulation [61, 62]. For individuals characterized by the P > C profile, alternative drivers of smoking relapse may be more relevant. With intact cognitive control and preserved natural‐reward responsiveness, individuals in this group may benefit more from strategies targeting withdrawal symptoms, stress, or negative affect. Behavioural activation (BA) could be a treatment well‐matched to this phenotype because BA aims at increasing engagement with non‐drug rewards [63, 64]. The profiles identified here therefore provide a rational basis for generating and testing mechanism‐informed treatment hypotheses in future trials, rather than a ready‐to‐use algorithm for treatment selection.

## 5 Conclusion

This study contributes to challenging a one‐size‐fits‐all model of addiction by identifying two neuroaffective profiles that reflect individual differences in the tendency to attribute incentive salience to drug‐related cues, a theoretically grounded mechanism of vulnerability to addictive behaviours. The profiles identified here differ not only in their neuroaffective responses to motivationally relevant stimuli, but also in the intrinsic functional connectivity of the fronto‐parietal control network (FPCN) at rest, with this resting‐state effect representing our primary connectivity finding. These converging task‐based and resting‐state results suggest two candidate neural pathways that may sustain addictive behaviours and, in future work, could be targeted by personalized interventions. Our strongest inferences rest on these effects, and additional subcortical–cortical connectivity differences observed in exploratory analyses should be considered hypothesis generating. Because our cross‐sectional data are correlational and did not directly assess impulse control or regulation in the presence of drug rewards, any interpretation of lower FPCN connectivity as a neural correlate of diminished control remains tentative and is based on prior literature. These results therefore lay the groundwork for future studies that will directly test this mechanism using task‐based measures of cognitive control and prospective relapse outcomes. Exploratory cross‐sectional correlations between ROI reactivity and clinical measures revealed several significant associations, which should be interpreted cautiously given the modest sample size. Future adequately powered studies should examine whether the magnitude of BOLD responses in key regions shows a dose–response relationship with clinical outcomes such as cue‐induced craving, time to relapse, or treatment response. Longitudinal studies incorporating reward‐predictive cues, reward‐related decisions, and treatment outcomes are required to further validate these profiles as predictive biomarkers and to clarify how network integrity contributes to the persistence of addiction across subtypes.

## Author Contributions

FV and BAT conceptualized and designed the study and secured funding. NS performed the fMRI data analyses and wrote the original draft of the manuscript. FV and BAT supervised the research and critically revised the manuscript. All authors reviewed and approved the final version of the manuscript.

## Funding

This work was supported by grant K23DA049216 from the National Institute on Drug Abuse (PI: B.A.T.), by grant P30CA016672 from the National Cancer Institute to the University of Texas MD Anderson Cancer Center and by generous philanthropic contributions to the Cancer Neuroscience Program of the University of Texas MD Anderson Cancer Center.

## Conflicts of Interest

The authors declare no conflicts of interest.

## Supporting information


**Figure S1:** Exploratory correlations between ROI reactivity (Cigarette − Pleasant) and clinical measures. Heatmaps display Spearman correlations for (A) the whole sample (*N* = 48), (B) the P > C profile (*n* = 26) and (C) the C > P profile (*n* = 22). Asterisks denote nominal significance (*p < 0.05, **p < 0.01 uncorrected). No correlations survived FDR correction for multiple comparisons.

## Data Availability

The data that support the findings of this study are available on request from the corresponding author. The data are not publicly available due to privacy or ethical restrictions.
