# Supplementary material for: Cortical and Subcortical Signatures of Incentive Salience Attribution in Tobacco Use Disorder
Source: Addict Biol. 2026 May 17;31(5):e70151. [Article in Spanish] doi: 10.1111/adb.70151 (PMC13181157; doi:10.1111/adb.70151)
Supplement: Supplementary file 1 — Figure S1: Exploratory correlations between ROI reactivity (Cigarette − Pleasant) and clinical measures. Heatmaps display Spearman correlations for (A) the whole sample (N = 48), (B) the P > C profile (n = 26) and (C) the C > P profile (n = 22). Asterisks denote nominal significance (*p < 0.05, **p < 0.01 uncorrected). No correlations survived FDR correction for multiple comparisons. [file ADB-31-e70151-s001.pdf]

SUPPELEMENTARY MATERIAL

Cortical and Subcortical Signatures of Incentive Salience Attribution in Tobacco Use Disorder

Nicola Sambuco[1], Francesco Versace[2], Brian A. Taylor[3]

[1] Department of Translational Biomedicine and Neuroscience, University of Bari Aldo Moro, Bari, Italy

[2] Department of Behavioral Science, The University of Texas MD Anderson Cancer Center, Houston, TX, USA

[3] Department of Imaging Physics, The University of Texas MD Anderson Cancer Center, Houston

Key words: fMRI, emotional pictures, tobacco use disorder, cue reactivity, individual differences, incentive salience, sign-tracking.

Correspondence to:
Nicola Sambuco, Ph.D., Department of Translational Biomedicine and Neuroscience, University of Bari Aldo Moro, Piazza Giulio Cesare 11, Bari, Italy, 70124. Telephone: +39 080 5593244. E-mail: nicola.sambuco@uniba.it
Francesco Versace, Ph.D., Department of Behavioral Science, Unit 1330, The University of Texas M. D. Anderson Cancer Center, PO Box 301439, Houston, TX 77230-1439. Telephone: +1 713 745 7933; FAX: (713) 794 4730. E-Mail: fversace@mdanderson.org

Exploratory Correlations Between Regional BOLD Reactivity and Clinical Measures

To examine potential level-dependent relationships between neural reactivity and clinical characteristics, we computed Spearman correlations between ROI-level BOLD responses (cigarette minus pleasant contrast) and available clinical measures. Change scores were extracted from three regions implicated in the Group × Content interaction: the amygdala–hippocampal complex (Amy/Hippo), left dorsolateral prefrontal cortex (lDLPFC), and medial prefrontal cortex (mPFC). Clinical measures included nicotine dependence (FTND), craving (QSU total, Factor 1, Factor 2), mood symptoms (PHQ-9, GAD-7), affect (PANAS positive and negative), anhedonia (SHAPS), trait impulsivity (BIS-11 attentional, motor, and nonplanning subscales), and smoking quantity (cigarettes per day). Correlations were computed for the whole sample ($N = 48$) and separately within each neuroaffective profile (P>C, $n = 26$; C>P, $n = 22$). Given the exploratory nature of these analyses, we applied Benjamini-Hochberg false discovery rate (FDR) correction within each sample.

Results are displayed in Supplementary Figure 1. In the whole sample, greater Amy/Hippo reactivity to cigarette versus pleasant cues was associated with higher anhedonia (SHAPS: $\rho = 0.31$, $p = .032$ uncorrected). Within the P>C profile, this association was stronger ($\rho = 0.56$, $p = .003$) and Amy/Hippo reactivity was also correlated with cigarettes per day ($\rho = 0.58$, $p = .005$). Additionally, lDLPFC reactivity showed an inverse relationship with motor impulsivity ($\rho = -0.46$, $p = .019$), while mPFC reactivity correlated positively with depressive symptoms (PHQ-9: $\rho = 0.44$, $p = .026$). Within the C>P profile, Amy/Hippo and lDLPFC reactivity were associated with relief-oriented craving (QSU Factor 2: $\rho = 0.45$, $p = .038$ and $\rho = 0.44$, $p = .042$, respectively), and mPFC reactivity correlated with motor impulsivity ($\rho = 0.43$, $p = .045$).

However, none of these correlations survived FDR correction (all $q > .09$). The strongest effects, observed in the P>C profile (Amy/Hippo with SHAPS and cigarettes/day), yielded FDR-corrected q-values of .093, approaching but not reaching the conventional threshold of $q < .05$. Given the modest sample sizes, the number of comparisons conducted (39 tests per sample), and the absence of FDR-corrected significance, these findings should be considered preliminary and hypothesis-generating. They parallel the exploratory subcortical–cortical connectivity results reported in the main text, which similarly reached nominal but not corrected significance. Future studies with larger samples and prospective clinical outcomes are needed to determine whether the magnitude of regional BOLD reactivity reliably predicts addiction severity or treatment response.

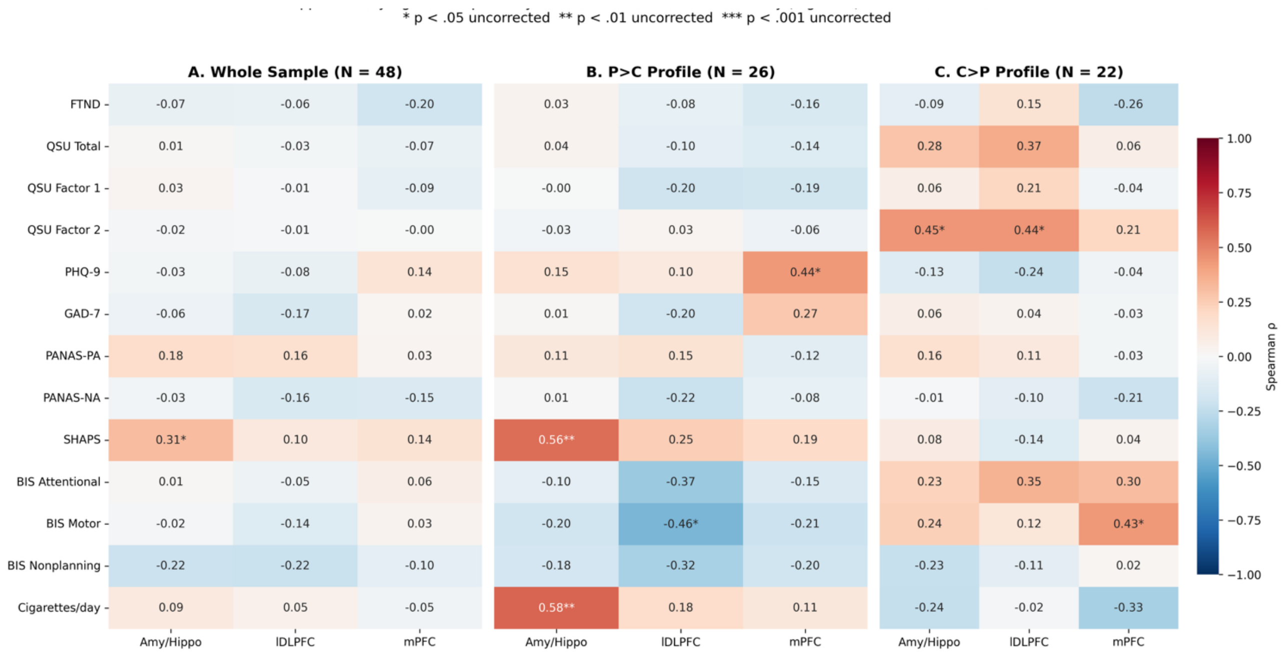

Supplementary Figure 1. Exploratory correlations between ROI reactivity (Cigarette minus Pleasant) and clinical measures. Heatmaps display Spearman correlations for (A) the whole sample (N = 48), (B) the P>C profile (n = 26), and (C) the C>P profile (n = 22). Asterisks denote nominal significance (*p < .05, **p < .01 uncorrected). No correlations survived FDR correction for multiple comparisons.
